# Bandwidth-Based Wake-Up Radio Solution through IEEE 802.11 Technology

**DOI:** 10.3390/s21227597

**Published:** 2021-11-16

**Authors:** Elena Lopez-Aguilera, Eduard Garcia-Villegas

**Affiliations:** Department of Network Engineering, Universitat Politècnica de Catalunya, 08034 Barcelona, Spain; elopez@entel.upc.edu

**Keywords:** IEEE 802.11, Wake-up Radio, green networks

## Abstract

IEEE 802.11 consists of one of the most used wireless access technologies, which can be found in almost all consumer electronics devices available. Recently, Wake-up Radio (WuR) systems have emerged as a solution for energy-efficient communications. WuR mechanisms rely on using a secondary low-power radio interface that is always in the active operation mode and is in charge of switching the primary interface, used for main data exchange, from the power-saving state to the active mode. In this paper, we present a WuR solution based on IEEE 802.11 technology employing transmissions of legacy frames by an IEEE 802.11 standard-compliant transmitter during a Transmission Opportunity (TXOP) period. Unlike other proposals available in the literature, the WuR system presented in this paper exploits the PHY characteristics of modern IEEE 802.11 radios, where different signal bandwidths can be used on a per-packet basis. The proposal is validated through the Matlab software tool, and extensive simulation results are presented in a wide variety of scenario configurations. Moreover, insights are provided on the feasibility of the WuR proposal for its implementation in real hardware. Our approach allows the transmission of complex Wake-up Radio signals (i.e., including address field and other binary data) from legacy Wi-Fi devices (from IEEE 802.11n-2009 on), avoiding hardware or even firmware modifications intended to alter standard MAC/PHY behavior, and achieving a bit rate of up to 33 kbps.

## 1. Introduction

More than two decades after the first IEEE 802.11 specification saw the light of day, the devices using that technology are counted in billions, and it continues to gain momentum [1]. IEEE 802.11 (or Wi-Fi) devices are nowadays present in almost all types of applications and appliances where network connectivity is required. However, many of the scenarios where we find Wi-Fi in use today are far out of the scope of the original specification, focused on the provision of broadband internet access to computers in a Wireless Local Area Network (WLAN). In this regard, the IEEE P802.11 Working Group has continued to improve this technology in an attempt to keep meeting the requirements of always-evolving user demands and to enable new scenarios and use cases. We can consider Wireless Sensor Networks (WSN) and the Internet of Things (IoT) as two of those new scenarios where Wi-Fi is trying to find its niche.

Those scenarios have their particular requirements; for example, WSN should cope with the failure of nodes, support mobility, and enable device-to-device communications through a multi-hop path [2], while IoT often requires the operation over long-distance point-to-multi-point wireless links. Nevertheless, both the IoT and WSN pose similar challenges to communication because (i) they have to provide support to a potentially large number of connected devices; (ii) most of the connected devices are resource-constrained (i.e., limited processing power, storage capacity); and (iii) devices have limited access to a power source.

Different amendments produced by IEEE P802.11 task groups offer partial support for those requirements. IEEE 802.11s, for example, supports reliable multi-hop transmissions at layer 2, enabling mesh-like topologies [3] to meet some of the requirements of WSNs. IEEE 802.11ah (certified under the name of Wi-Fi HaLow), on the other hand, focuses more on the IoT case.

Regarding the support of a large number of devices, IEEE 802.11ah allows more than 8000 connected stations (STAs); note that legacy Wi-Fi has a (theoretical) upper limit of 2007 STAs per access point (AP), which is impractical due to the high collision probability. Recall that legacy IEEE 802.11 defined a Carrier Sense Multiple Access scheme with Collision Avoidance (CSMA/CA); that is, an STA willing to transmit first senses the medium, if it is free, the STA starts transmitting one or more frames within a Transmission Opportunity (TXOP) of limited duration. If the channel is sensed to be busy, and before a new attempt to gain access to the medium, the STA sets a backoff timer, choosing a random number of slots between 0 and a given Contention Window value (CW). The backoff timer is paused while the medium stays busy and resumes when the medium is free. If the transmission fails, CW is doubled. Despite these measures, the performance of legacy Wi-Fi networks degrades drastically in the presence of hundreds, even tens of simultaneous transmitting STAs. To minimize the impact of collisions in denser scenarios, IEEE 802.11ah also introduces a new channel access mechanism called Restricted Access Window (RAW). RAW divides STAs into different groups and restricts channel access only to a group during a particular time period, reducing the number of simultaneous contenders and, thus, reducing collisions [4]. This amendment also extends coverage by allowing more robust Modulation and Coding Schemes (MCS) and using a lower frequency band below 1 GHz, while most Wi-Fi networks use either 2.4 GHz (case of IEEE 802.11b/g/n/ax) or 5 GHz (case of 11a/n/ac/ax) Industrial, Scientific and Medical (ISM) bands. IEEE 802.11ah also extends range by allowing signals of different bandwidths (i.e., concentrating energy over a narrower band, increases Signal to Interference and Noise Ratio (SINR)) from 1 to 16 MHz, while legacy Wi-Fi uses 20 MHz of bandwidth. IEEE 802.11n, on the other hand, allows transmissions of 20 or 40 MHz, and IEEE 802.11ac and 11ax add the options of 80 and 160 MHz, which can be set on a per-packet basis.

Power saving is another issue that has been considered since the initial IEEE 802.11 standards, which allowed STAs to remain in a Power Saving Mode (PSM) to reduce energy consumption and extend battery time. In PSM, inactive STAs are allowed to move to an energy-saving state (i.e., go to sleep). Meanwhile, the serving AP buffers downlink frames addressed to sleeping STAs. Those STAs wake up periodically to check whether they have pending frames; if not, they can go back to sleep; if yes, STAs poll the AP to retrieve those frames and return to the power-saving state. In the legacy PSM, STAs could remain inactive for several hours, whereas IEEE 802.11ah allows longer periods of sleep (to the year scale) [4]. IEEE 802.11ah also introduces the Target Wake-up Time (TWT) mechanism, by which the AP and STAs agree on a specific serving period, thus reducing the time an STA must be awake to check for downlink frames. In a similar way, the RAW mechanism helps in saving energy as well by allowing STAs to sleep outside their assigned transmission window. These power-saving mechanisms, however, suffer from two sources of inefficiency: (i) STAs must wake up periodically, many times unnecessarily if there is no pending traffic for them; and (ii) time-sensitive frames have to wait for possibly long times in AP’s buffers before the recipient STA wakes up from PSM, or at TWT.

Those inefficiencies can be avoided by the use of Wake-Up Radio (WuR) mechanisms [5]. In a WuR system, energy-constrained networking devices are equipped with a secondary low-power radio, which is always awake, while the main radio is allowed to sleep for long periods. In the presence of downlink traffic, the transmitter sends a Wake-up Call (WuC) addressed to the recipient STA’s secondary radio, which will immediately wake up the primary radio in order to start receiving those frames with a minimum delay. IEEE 802.11ba defines such a system for Wi-Fi 6 devices and beyond, as they are not backward compatible with previous generations of Wi-Fi devices, not even with Wi-Fi 6 devices already available on the market. In this paper, however, we define a novel WuR solution, which enables any existing Wi-Fi device capable of transmitting different bandwidths (i.e., from IEEE 802.11n-2009 on) to become a WuR Transmitter (WuTx). We also propose a simple Wake-up Receiver (WuRx) design, which we show, through a comprehensive set of Matlab simulations, that represents a suitable and feasible IEEE 802.11-based WuR solution.

The remainder of this paper is structured as follows. Section 2 presents with more detail the concept of WuR and reviews existing literature on IEEE 802.11-based WuR applications. Section 3 describes our proposed solution for a WuR using IEEE 802.11 transmitters, while Section 4 discusses an exemplary implementation of a compatible WuRx. Section 5 proves the feasibility of our approach by means of a simulation-based study, and Section 6 provides concluding remarks.

## 2. State of the Art on IEEE 802.11-Based Wake-Up Radio Solutions

**Figure 1:**
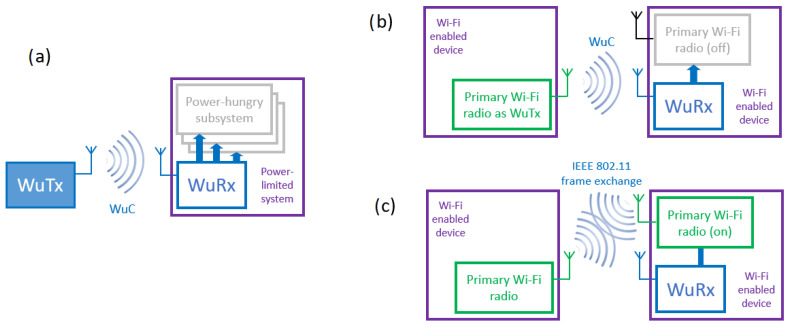
(**a**) Generic Wake-up Radio (WuR) system, where a Wake-up Transmitter (WuTx) sends a Wake-up Call (WuC) to a Wake-up Receiver (WuRx); (**b**) Wi-Fi-based WuR system where a Wi-Fi radio is used as a WuTx; (**c**) Wi-Fi-based WuR where primary radios are used for a regular data frame exchange.

In general, any WuR system can be depicted by the scheme in Figure 1a, where a power-limited device is required to save energy by disabling one or more power-hungry subsystems. Said device is equipped with a WuRx, a simple radio receiver circuit (i.e., no transmission capability) designed to have a very low energy consumption. When those inactive subsystems are required to resume their normal operation, a particular radio signal, called WuC, is transmitted from the WuTx to the WuRx, which will wake up those subsystems from their power-saving state. The WuRx can be as simple as a capacitor and a rectifying diode [6]. More sophisticated WuRx designs include active elements, offering additional features, such as the possibility to send WuC to a specific WuRx or group of receivers (i.e., addressable WuC) or the capacity to decode and process binary data embedded in the WuC. Still, even with the presence of active elements, a WuRx requires power in the scale of µW, or even nW [7].

In the case of IEEE 802.11-based WuR, the Wi-Fi radio is considered as the primary radio used for the regular exchange of data frames, which benefits from the security features, and high transmission rates supported by the IEEE 802.11 specifications. However, although highly efficient in terms of energy per bit [8], Wi-Fi radios show high power consumption figures, even in an idle state (i.e., neither transmitting nor receiving). In an idle state, STAs must always be ready to detect known preambles (signaling the start of a frame), and this implies a power consumption in the scale of hundreds of mW. It is, therefore, a good idea to put that primary radio to sleep while a very low-power radio (the WuRx or secondary radio) remains active, ready to wake up the primary radio upon request. When a transmitter needs to deliver IEEE 802.11 frames to the sleeping STA, it will buffer those frames and send a WuC.

Solutions like the one in [9] use out-of-band signaling for the WuC, which requires a secondary radio also at the transmitter side. This implies that (i) primary and secondary radios may operate at different distances (e.g., the WuC reaches the WuRx, but then, communications using the primary radio are not possible at that distance), and (ii) the need for a dedicated secondary radio to work as WuTx limits the applicability of such solutions. For those reasons, in our approach, we build the WuC from an IEEE 802.11-compliant interface, which is used both as WuTx and as the primary radio for high-rate data transfers, as shown in Figure 1b,c. In Figure 1b, the concept of WuR presented in Figure 1a is applied for the usage of the IEEE 802.11-compliant interface as WuTx and, thus, the WuC is built from legacy IEEE 802.11 transmissions. Figure 1c shows an example in which the primary interface in the receiver side is awake after the WuRx has received the IEEE 802.11-based WuC, and IEEE 802.11 primary interfaces at transmitter and receiver can start exchanging regular data frames. As discussed in [10], an IEEE 802.11-based WuTx enables the implementation of multiple applications using the concept of WuR shown in Figure 1a, given that Wi-Fi hardware is easily available at low costs.

Different WuR systems have been studied in the literature, where researchers present different solutions that can use a Wi-Fi device (with modifications or completely off-the-shelf) as WuTx [10]. According to how IEEE 802.11 signals are used to generate the WuC, we classify these WuR systems into two groups [11]: (i) systems that use sequences of standard IEEE 802.11 frames to encode the WuC; and (ii) systems that encode the WuC within IEEE 802.11 Orthogonal Frequency-Division Multiplexing (OFDM) signals at the symbol level. The first type of system uses standard IEEE 802.11 frame transmissions and, therefore, are compatible with off-the-shelf Wi-Fi devices with only a software update. On the other hand, the WuC bit rate offered by these systems is low, limited by the achievable frame rate (note that these systems can encode, at most, one WuC symbol per IEEE 802.11 frame). Besides, WuCs comprising long sequences of frames can be broken by interfering transmissions due to the random access nature of the IEEE 802.11 MAC. In [12], for example, a simple, non-addressable WuC is encoded by the transmission of any IEEE 802.11 frame; the WuRx will wake up the device upon detecting any energy above a given threshold in a Wi-Fi channel. The solution is easy to implement but is prone to frequent false detections in the presence of other transmissions in the same band (e.g., Wi-Fi, Bluetooth, etc.). The same authors extend this idea to send a sequence of frames, combined with periods of silence to encode binary data (e.g., an address) [13]. In [14], WuC symbols are encoded using the length of IEEE 802.11 data frames, but the WuRx design consumes power in the order of the mW. Authors in [15] presented a μW order WuR system wherein an On-Off Keying (OOK) signal is emulated by combining silent periods, representing OFF symbols, and ON symbols generated by the transmission of successive IEEE 802.11 frames of minimum length. Due to the large WuC symbol duration, the effective bit rate of this solution is lower than 1 kbps. Reference [10] employs the WuR system of [15] and presents a proof-of-concept smart plug system implementation for a use case consisting of a green Wi-Fi application.

WuR solutions of the second type (i.e., IEEE 802.11’s OFDM symbol level), on the other hand, allow a higher WuC bit rate, leveraging 250 kBd of the IEEE 802.11’s OFDM PHY using 4 μs symbols. Authors in [16] manipulate OFDM symbols, forcing some subcarrier sets to zero, thus encoding the WuC’s binary data in the frequency domain. Similarly, authors in [17] modulate the WuC using OOK by activating/deactivating the central subcarriers of an IEEE 802.11g/n OFDM signal. The system in [17] was integrated using 14-nm FinFET CMOS technology and consumed less than 100 μW. A system using the symbols of an IEEE 802.11ah 256-QAM constellation is presented in [18], the implementation of which was tested on a software-defined radio platform. Those approaches present a common drawback in their implementation complexity since they require low-level access to PHY functions (e.g., direct access to I/Q samples), which is not supported by legacy Wi-Fi interfaces, thus preventing its use from billions of already existing devices [2]. This drawback, however, is partially overcome by the solution proposed in [11]. In [11], authors reverse-engineer the full IEEE 802.11’s OFDM PHY to generate specific data bit sequences at the output of the MAC layer, which will translate into the wanted OFDM symbols at the output of the PHY. In this case, a simple software application can be used to inject those bit sequences through common raw socket interfaces. This approach, however, requires prior knowledge of the random seed used by the scrambler block at the input of the PHY, something manufacturers are reluctant to share.

In light of the growing interest in this topic, the IEEE P802.11WG started to work on standardizing a WuR solution and created the TGba task group. TGba’s IEEE 802.11ba specification was released in October 2021 [19]. In fact, different receiver designs, compatible with the current specification, have already been published [20,21]. The IEEE 802.11ba also operates at the OFDM symbol level and uses OOK to modulate WuC data [10,22]. Following the same principles defined in [17], IEEE 802.11ba WuC follows a 20 MHz non-High Throughput (non-HT) preamble, used to allow legacy STAs to detect the WuC and, thus, prevent collisions. After the legacy preamble, the WuC is generated, activating/deactivating the central 13 subcarriers of a 20 MHz IEEE 802.11’s OFDM symbol, thus reducing the effective bandwidth to 4 MHz. The WuC contains a synchronization sequence intended to allow frame detection and synchronization by the WuRx, and the WuC data, which is Manchester encoded. Two different encodings are defined: 1 WuC bit per IEEE 802.11 OFDM symbol (i.e., 1 bit per 4 μs), yielding 250 kbps, and 1 WuC bit per 4 OFDM symbols (i.e., 1 bit per 16 μs), offering 62.5 kbps.

Our goal is to offer an IEEE 802.11-based WuR solution that is compatible with the existing ecosystem of Wi-Fi devices at the cost of a minimum software update. The only requirement for a Wi-Fi transmitter to work as WuTx is the support of channel bonding (available since IEEE 802.11n-2009 [23]). Therefore, our proposal is based on the first type of system, that is, WuR systems based on the transmission of standard IEEE 802.11 frames, increasing over the effective bit rate provided by [10,15], and not requiring the manipulation of OFDM symbols at the PHY as the one shown in [11]. As detailed in the following sections, our solution minimizes the limitations of these types of systems by (i) using the shortest possible frame to maximize the effective WuC bit rate, and (ii) transmitting the sequence of frames comprising the WuC within one TXOP, hence protecting the whole sequence against interruptions by other Wi-Fi STAs in the area.

## 3. Bandwidth-Based Wake-Up Radio Solution through IEEE 802.11

**Figure 2:**
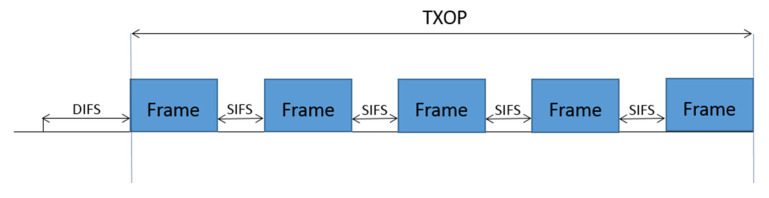
Example of a standard-compliant transmission using a Transmission Opportunity (TXOP).

The WuR system proposal presented in this paper is based on the transmission of legacy frames by an IEEE 802.11 standard-compliant transmitter during a TXOP period. According to the standard specification, after the transmitter gains access to the medium following the IEEE 802.11 MAC contention mechanism, frames are transmitted consecutively within a TXOP, being those frames separated from each other by a Short Inter-Frame Space (SIFS) interval (Figure 2). Note that the number of frames involved in the TXOP of Figure 2 are only for the sake of example; in practice, any number of frames fitting within the TXOP limit is allowed. Moreover, the proposal also exploits the PHY characteristics of specifications from IEEE 802.11n on, where different signal bandwidths can be employed for frame transmission, making use of the so-called channel bonding mechanism. In this way, IEEE 802.11n allows operation for 20 and 40 MHz of signal bandwidth, while IEEE 802.11ac and 11ax can use 20, 40, 80, and 160 MHz. If transmitted frames inside a TXOP employ MCSs using different signal bandwidths, and these signals of different bandwidths can be distinguished at the receiver, the bandwidth of such transmitted frames can be used to encode different symbols composing a WuC. That is, the information transmitted to the WuRx is not in the payload contained in those IEEE 802.11 frames but in the bandwidth used to transmit those frames. Such a solution will allow an IEEE 802.11 standard-compliant transmitter to be used as WuTx for WuC generation, while the corresponding reception and decoding at WuRx do not need to implement the complexity of an IEEE 802.11 receiver; a simple receiver capable of distinguishing transmissions of different bandwidth can be used as WuRx. Moreover, corresponding coded symbols in a WuC will be intrinsically protected in front of other WuCs and of IEEE 802.11 legacy transmissions through the usage of the TXOP.

### 3.1. Wake-Up Radio Transmitter and Wake-up Radio Call

**Figure 3:**
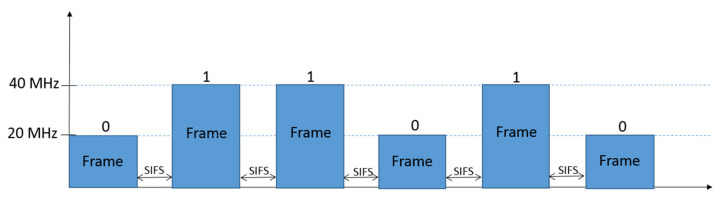
Example of one bit per symbol coding with two different signal bandwidths. A frame with 20 MHz of signal bandwidth represents bit “0”, and a frame using 40 MHz the bit “1”.

**Figure 4:**
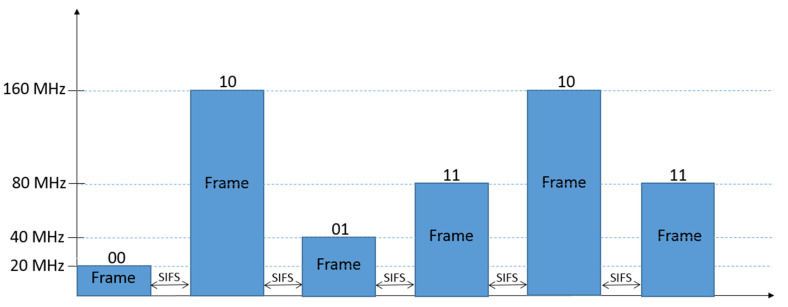
Example of two bits per symbol coding with four different signal bandwidths. A frame with 20 MHz of signal bandwidth representing bits “00”, a frame using 40 MHz represents bits “01”, 80 MHz corresponds to “11”, and 160 MHz to “10”.

For the WuTx proposed in this research, first, a legacy IEEE 802.11n transmitter can be used, sending frames during a TXOP employing two different signal bandwidths, thus coding two different symbols, each of them composed by one bit (cf. Figure 3). On the other hand, for an IEEE 802.11ac/ax transmitter, up to four different bandwidths for frame transmission during a TXOP can be used, thus allowing four different symbols, each of them carrying two bits (cf. Figure 4). Obviously, WuTx with the IEEE 802.11ac/ax option also allows the one bit per symbol coding of Figure 3, but other combinations using two bandwidth values could be used to increase the distance between symbols (e.g., 20 and 80 MHz, or 20 and 160 MHz). Note that the number of frames involved in the TXOP of Figure 3 and Figure 4 are only for the sake of example.

IEEE 802.11 frames used in the proposed WuC are composed of PHY preamble and header and MAC header (i.e., empty data payload), thus consisting in IEEE 802.11 standard-compliant frames of minimum length available for transmission. The fastest MCS is chosen for the different signal bandwidths in order to minimize frame duration (i.e., number of OFDM symbols) employing one spatial stream. Thus, for 20 MHz of signal bandwidth, the minimum duration for an IEEE 802.11n transmission is 44 µs (36 µs for PHY preamble and header and 8 µs for MAC header), whereas, for IEEE 802.11ac, it is 48 µs (40 µs for PHY preamble and header and 8 µs for MAC header). On the other hand, for higher values of signal bandwidth (i.e., 40, 80, and 160 MHz), MAC header duration can be reduced to the minimum value, i.e., one OFDM symbol of 4 µs. This leads to a frame duration of 40 µs for 40 MHz with IEEE 802.11n and of 44 µs for 40, 80, and 160 MHz with IEEE 802.11ac. With regard to IEEE 802.11ax, the minimum frame duration employing signal bandwidths from 20 to 160 MHz is 57.6 µs (44 µs for PHY preamble and header, and 13.6 µs for MAC header). Reference [24] shows detailed computation on frame transmission times for the different IEEE 802.11 amendments and configurations.

Following IEEE 802.11 standard specification, consecutive frames within a TXOP are separated from each other by a SIFS interval, which is set to 16 µs for IEEE 802.11ac and IEEE 802.11n/ax amendments operating on the 5 GHz frequency band. For IEEE 802.11n/ax using 2.4 GHz spectrum, the SIFS value is 10 µs; however, a signal extension of 6 µs has to be added at the end of each frame, thus becoming 16 µs the total amount of time between frames. Taking this into account, a symbol duration of 60 µs is obtained when using IEEE 802.11n signals of 20 MHz and one bit per symbol (i.e., 44 µs of frame duration plus 16 µs of inter-frame interval), thus leading to a bit rate of 16.67 kbps. For IEEE 802.11n signals of 40 MHz and one bit per symbol, a bit period of 56 µs and, consequently, a bit rate of 17.85 kbps is achieved. Thus, an average bit rate of 17.26 kbps is obtained when using IEEE 802.11n to encode a bandwidth-based WuC with two symbols. Bit rate is slightly reduced when using an IEEE 802.11ac transmitter and one bit per symbol (15.63 kbps for signals of 20 MHz and 16.67 kbps for signals larger than 40 MHz, i.e., 16.15 kbps on average). For IEEE 802.11ax, the achieved bit rate is 13.58 kbps. On the other hand, bit rates are obviously doubled for the scenario with two bits per symbol, leading for the case of IEEE 802.11ac WuTx to 31.25 kbps for signals of 20 MHz and to 33.33 kbps for signals of 40, 80, or 160 MHz, i.e., 32.81 kbps on average. For the case of IEEE 802.11ax, the bit rate is increased to 27.17 kbps for all the available signal bandwidths. The average symbol rate is expected to be used as the sample rate in the WuRx for symbol decoding, with synchronization guaranteed for typical WuC lengths of around 16 bits [9,13,14,15,25]. Alternatively, for longer WuC length and in order to avoid synchronization issues, we can force different IEEE 802.11n/ac symbols to have the same duration, applying an extension of 4 µs to signals larger than 20 MHz by the usage of bit padding in the MAC payload.

### 3.2. Wake-Up Radio Receiver

**Figure 5:**
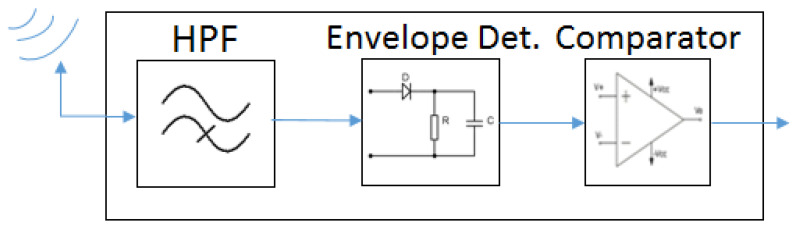
Main composition of WuRx.

As stated, the proposed Wake-up Radio system works with a simple WuRx design, provided that it is capable of distinguishing transmissions using different bandwidths in Wi-Fi frequencies (i.e., 2.4, 5 GHz bands). The WuRx presented in this paper is composed of three components: High-Pass Filter (HPF), envelope detector, and comparator (Figure 5). The HPF is the key component of this WuRx, as it is responsible for filtering the incoming signal in order to distinguish among the different signal bandwidths. OFDM signals with different bandwidth values contain a different number of subcarriers. In the case of IEEE 802.11n and 11ac, for example, 20 MHz signals are built of 64 subcarriers, 40 MHz signals of 128 subcarriers, 80 MHz signals of 256 subcarriers, and 160 MHz signals of 512 subcarriers, containing the signals with larger bandwidth also the subcarriers of the signals with shorter bandwidth (i.e., the 512 subcarriers of the 160 MHz signal include the 64 subcarriers of the 20 MHz, the 128 subcarriers of the 80 MHz signal, and the 256 subcarriers of the 80 MHz signal). Given the aforementioned characteristics of OFDM signals, the usage of an HPF with the appropriate cut-off frequency will allow the differentiation among signals with different bandwidths, i.e., a different number of subcarriers. In this way, for instance, a 20 MHz signal can be filtered using a cut-off frequency above the frequency components of its 64 subcarriers, whereas signals with larger bandwidth values will still maintain high signal levels at this filter output.

After filtering, the signal is handled by the envelope detector block in order to convert the signal to a DC voltage. Finally, the output of the envelope detector is introduced in the comparator block and compared with a pre-defined threshold value. Based on this comparison, the signal bandwidth of the incoming signal at the receiver input is decided, thus determining the symbol and corresponding bit values composing the WuC. For instance, after filtering using a cut-off frequency above the frequency components of 64 subcarriers, if the signal at the input of the comparator block is below the threshold, the decision is that a 20 MHz signal is received; otherwise, it is decided that a signal above 20 MHz is received. Note that an initial pre-defined preamble will be included at the beginning of the WuC, thus indicating the start of the WuC frame, followed by the sequence of coded bits (i.e., address or other binary data). The aforementioned process and WuRx illustrated in Figure 5 are feasible for distinguishing between two signal bandwidths, and thus, for the scenario employing two different symbols and one bit per symbol. Besides the elements shown in Figure 5, additional hardware will be necessary for the identification of the address or of the binary data received (e.g., data slicer and address correlator); however, this part is out of the scope of the present research, and we leave it for future work.

**Figure 6:**
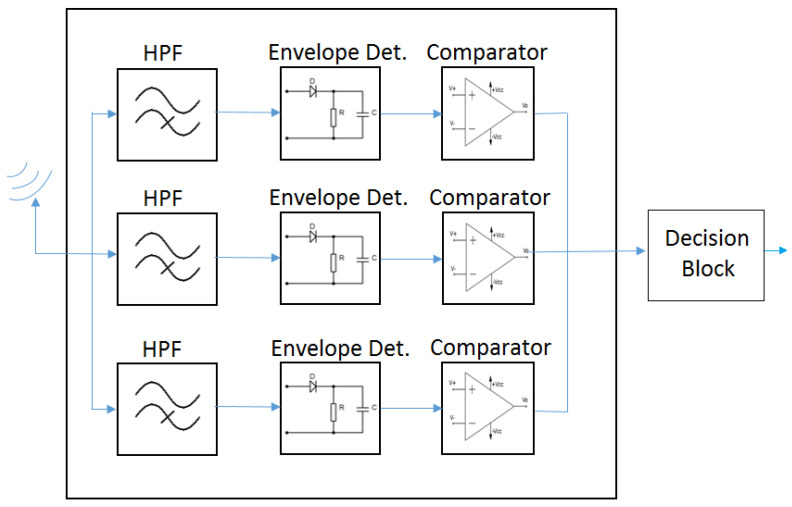
WuRx for two bits per symbol.

**Figure 7:**
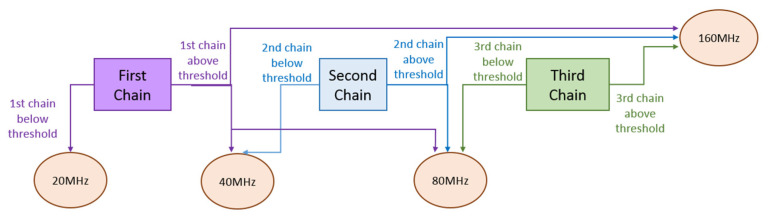
Decision flow performed at the WuRx.

For the scenario with four different symbols and two bits per symbol, the WuRx of Figure 5 is insufficient to distinguish among signals with four different bandwidth values. Thus, the WuRx of Figure 6 is proposed, which includes three receiver chains as the one shown in Figure 5 and a logical decision block. The incoming signal is handled by each receiver chain, being the differences among chains, the cut-off frequency of the filters, and the threshold value of the comparator blocks. The objective of the first filter and chain consists in distinguishing between a 20 MHz signal and signals with larger bandwidth values. The second filter and chain aim at differentiating between 40 MHz signals and signals above 40 MHz of bandwidth. The third filter and chain target is to discriminate between an 80 MHz signal and a 160 MHz signal. Finally, the outputs of these three chains consist of the inputs of the logical decision block, which takes the decision on the signal received and, thus, on the symbol and corresponding bit decoding. If the output of the first chain is below the corresponding threshold, the decision is that a 20 MHz signal is received, regardless of the output of the second and third chains. On the other hand, if the output of the first chain is above the corresponding threshold, and the output of the second chain is below the threshold, then the decision is that a 40 MHz signal is received, regardless of the output of the third chain. If the outputs of the first and second chains are above the corresponding thresholds and the output of the third chain is below the threshold, the decision is that an 80 MHz signal is received. Finally, in case the outputs of the three chains are above the corresponding thresholds, then it is decided that a 160 MHz signal is received. The decision flow is illustrated in Figure 7. The choice of cut-off frequencies and threshold values (explained in the following Section 4) is crucial for the correct discrimination among signals of different bandwidths and, thus, for obtaining good WuRx performance with regard to WuC decoding.

## 4. Wake-Up Radio Receiver Design

To carry out the design of the WuRx described in Section 3.2, we have used the Matlab software tool. Moreover, we have used functions from the WLAN System Toolbox for the generation of IEEE 802.11 frames and their transmission through a communication channel, taking into account the influence of multi-path and fading. Propagation models from TGn/ac [26] have been employed.

**Figure 8:**
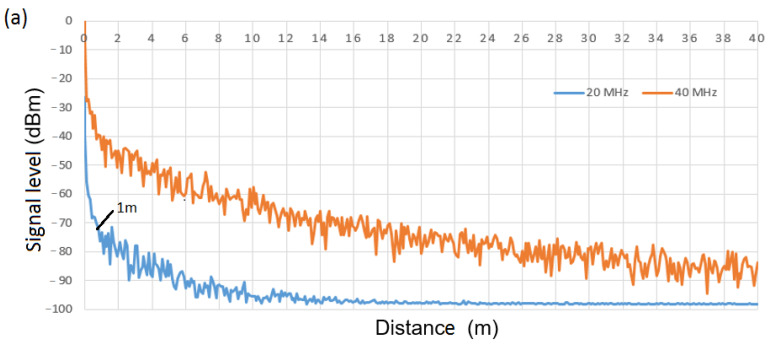

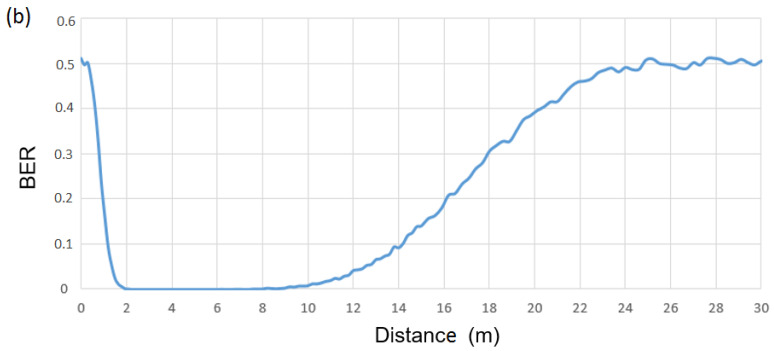
(**a**) Signal level at filter output of Figure 5, (**b**) Bit Error Rate (BER) at WuRx output of Figure 5, vs. distance, employing 20 MHz and 40 MHz signals.

For the WuRx design, it is necessary to decide on the thresholds of the different comparator blocks. For this issue, we consider propagation model B from [26], as it is the most restrictive (model B applies to indoor, residential, or small office environments). We begin with the WuRx design of Figure 5 (scenario with two symbols and one bit per symbol) and choose a fifth-order Chebyshev filter with a cut-off frequency of 12 MHz. This filter configuration has been chosen to filter a 20 MHz signal using the lowest filter order providing enough attenuation to differentiate between a 20 MHz IEEE 802.11 signal and a signal with a larger bandwidth. The signal level at filter output for a 20 MHz and a 40 MHz IEEE 802.11 signal is shown in Figure 8a as a function of the distance between transmitter and receiver. Note that if differentiation between a 20 MHz and a 40 MHz signal is successfully achieved, it is also feasible between a 20 MHz signal and a signal with bandwidth larger than 40 MHz (i.e., 80 MHz and 160 MHz). A transmission power of 1 W is considered at 5 GHz. From this figure, we observe around 30 dB of difference between output signals at 1 m of distance and, thus, we establish the 20 MHz signal level at 1 m (i.e., −73 dBm) as the threshold value at the comparator block. This means that a received IEEE 802.11 signal with a signal level at the filter output below this threshold will be decoded as a 20 MHz signal; otherwise, it will be decoded as a 40 MHz signal, thus leading to an effective operational range covering distances from 1 m to 12 m. If we consider a stream of 10,000 random bits, where IEEE 802.11 frames of 20 MHz of bandwidth encode WuC bits with a value of “0”, and frames of 40 MHz of bandwidth encode bits with a value of “1”, Figure 8b shows corresponding Bit Error Rate (BER) at the output of WuRx of Figure 5. As shown, BER = 0 is achieved in the range between 1 m and around 12 m. Choosing a lower threshold value would improve the performance in short distances (i.e., <1 m), however, it will also decrease the effective range below 12 m. In this way, we choose a lower bound of 1 m, which we found reasonable for the WuR use case, where the aim is the management of remote devices beyond 1 m.

**Figure 9:**
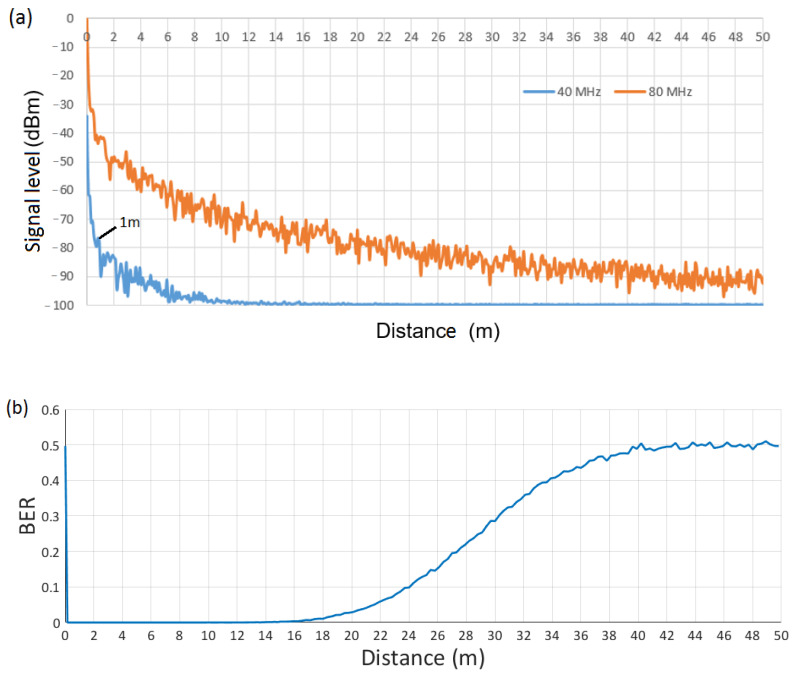
(**a**) Signal level at filter output of the second chain of Figure 6, (**b**) BER at the output of the second chain of the WuRx of Figure 6, vs. distance, employing 40 MHz and 80 MHz signals.

**Figure 10:**
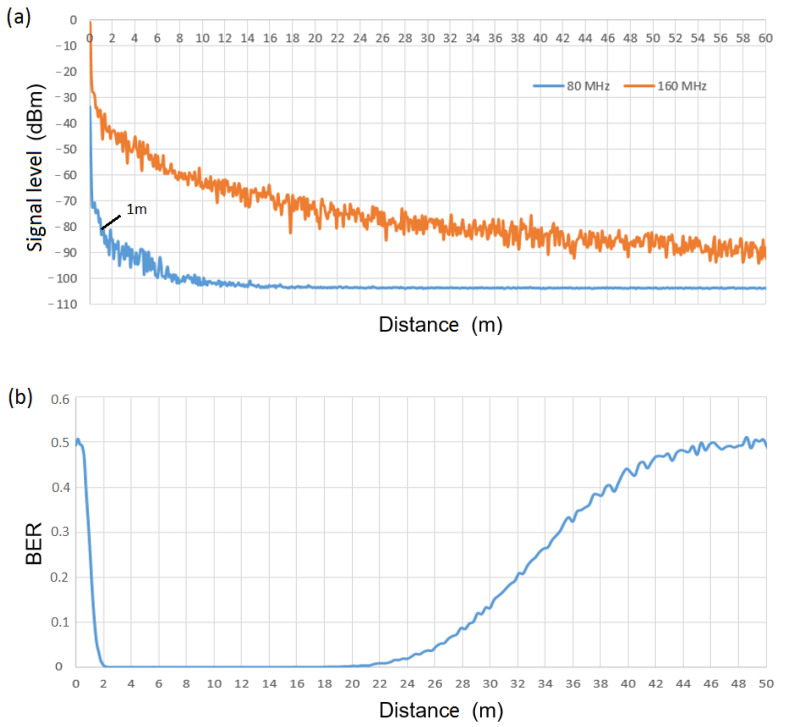
(**a**) Signal level at filter output of the third chain of Figure 6, (**b**) BER at the output of the third chain of the WuRx of Figure 6, vs. distance, employing 80 MHz and 160 MHz signals.

With regard to the WuRx of Figure 6 (scenario with four symbols and two bits per symbol), threshold values for the comparator blocks of second and third chains have to be defined (note that the aforementioned threshold for WuRx of Figure 5 is employed in the first chain of the WuRx of Figure 6). Following the same approach, we choose a fourth-order Chebyshev filter and cut-off frequency of 33 MHz in the second chain to filter a 40 MHz signal using the lowest filter order providing enough attenuation to differentiate between a 40 MHz IEEE 802.11 signal and a signal with a larger bandwidth. Finally, a third-order Chebyshev filter and cut-off frequency of 63 MHz is chosen in the third chain to filter an 80 MHz signal. The signal level at filter output of the second chain for a 40 MHz and an 80 MHz IEEE 802.11 signal, as a function of the distance between transmitter and receiver, is observed in Figure 9a. Note that if differentiation between a 40 MHz and an 80 MHz signal is successful, then it is also suitable with signals of bandwidth larger than 80 MHz (i.e., 160 MHz). A value of −77 dBm is chosen as the threshold at the comparator block (i.e., 40 MHz signal level at 1 m), leading to an operational range (BER = 0) from 1 m to 18 m. Corresponding BER at the output of the second chain of the WuRx of Figure 6 is shown in Figure 9b. A stream of 10,000 random bits is considered, where IEEE 802.11 frames of 40 MHz of bandwidth encode WuC bits with a value of “0”, and frames of 80 MHz of bandwidth encode bits with a value of “1”. Finally, Figure 10a shows the signal level at filter output of the third chain for an 80 MHz and a 160 MHz IEEE 802.11 signal, being −81 dBm chosen as a threshold value (i.e., 80 MHz signal level at 1 m) and resulting in an operational range (BER = 0) from 1 m to 22 m. This range can also be observed from Figure 10b, which shows BER performance at the output of the third chain of the WuRx of Figure 6, considering a stream of 10,000 random bits, with IEEE 802.11 frames of 80 MHz of bandwidth encoding WuC bits with a value of “0”, and frames of 160 MHz of bandwidth encoding bits with a value of “1”. It is important to highlight that we use the aforementioned threshold values for evaluation purposes. More than the absolute numbers obtained and used in this validation, what is crucial is to achieve enough power difference between signals of different bandwidths at the filters’ outputs. If the signal level at the input of the envelope detector needs to be above a minimum value, it can be achieved by adding an amplification step.

With four symbols and two bits per symbol, it is necessary to choose the codification scheme. In this way, we use the codification scheme shown in Table 1, where there is one bit of difference between adjacent symbols, i.e., between a 20 MHz and a 40 MHz signal, between a 40 MHz and an 80 MHz signal, and between an 80 MHz and a 160 MHz signal. This leads to better BER performance than using the coding scheme with bits “00”, “01”, “10” and “11” for a 20 MHz, a 40 MHz, an 80 MHz, and a 160 MHz signal, respectively. Note that, in this last case, there are two bits of difference between a signal of 40 MHz and a signal of 80 MHz. For the case with two symbols and one bit per symbol, a 20 MHz signal is always decoded as a bit of “0”, while a signal with higher bandwidth represents a bit of “1”.

## 5. Wake-Up Radio System Evaluation

In this section, we evaluate the proposed WuR system and compare the performance of the one bit per symbol and of the two bits per symbol WuC, considering legacy IEEE 802.11 transmitters.

Again, we have used the Matlab software tool and the functions from the WLAN System Toolbox for the generation of IEEE 802.11 frames and their transmission through a communication channel, including multi-path and fading effects. In addition to propagation model B, also model F from TGn/ac [26] has been used, which is employed for large open indoor and outdoor scenarios.

**Figure 11:**
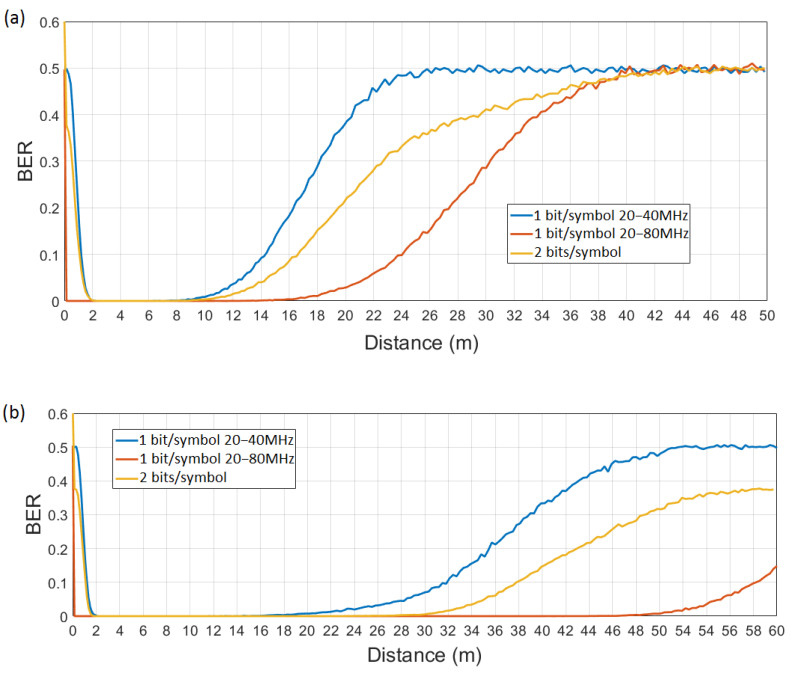
BER at WuRx output of Figure 6 vs. distance, for propagation model B (**a**) and F (**b**).

We consider a bit stream of 10,000 random bits for the one bit per symbol WuC and of 20,000 for the two bits per symbol WuC, thus, transmitting 10,000 IEEE 802.11 signals (i.e., WuC symbols) in both scenarios. Signals are generated according to the coding scheme described in Section 4. Obviously, IEEE 802.11n transmissions can only be employed for the one bit per symbol WuRx with 20 MHz and 40 MHz signal bandwidths since IEEE 802.11n does not support larger bandwidths. Moreover, note that, although the different IEEE 802.11 amendments (i.e., IEEE 802.11n/ac/ax) introduce some changes at the PHY, the generated signal for different bandwidths (i.e., 20, 40, 80, and 160 MHz) is read by the proposed WuRx of Section 4. Thus, independently of the specific PHY used at the WuTx, the WuR solution is suitable for the different amendments from IEEE 802.11n-2009 on, given that the spectral mask at different bandwidths is almost identical for all of them [23,27,28]. Recall that the WuRx is intended to detect energy over different bandwidths, which are common to all amendments operating in the 2.4 and 5 GHz ISM bands; that is, from IEEE 802.11n to IEEE 802.11ax (both included). A transmission power of 1 W is considered at 5 GHz; therefore, we only show the evaluation at 5 GHz using an IEEE 802.11ac WuTx. We take the different alternatives into account for our evaluation and show BER performance at WuRx output as a function of the distance between WuTx and WuRx in Figure 11a,b for propagation models B and F, respectively. The largest operational range (BER = 0), from 1 m to 18 m for model B and from 1 m to 50 m for model F, is achieved by the approach using one bit per symbol encoded with 20 MHz and 80 MHz signals. The scenario with one bit per symbol employing 20 MHz and 160 MHz signals shows the same performance as for the case including 20 MHz and 80 MHz signals and, thus, it has not been included in Figure 11. The system with two bits per symbol decreases the operational range (BER = 0) from a maximum distance of 18 m to 12 m for model B, and from a maximum of 50 m to 30 m for model F. On the other hand, it allows a higher bit rate (32.81 kbps in front of 16.14 kbps and 17.26 kbps, using one bit per symbol and IEEE 802.11ac and IEEE 802.11n, respectively; 27.17 kbps in front of 13.58 kbps for IEEE 802.11ax). The worst operational range (up to a maximum distance of around 10 m for model B, and up to a maximum of 20 m for model F) is achieved for one bit per symbol and signal bandwidths of 20 MHz and 40 MHz. Slope values are smoother for the approach with better performance (1 bit/symbol 20–80 MHz), i.e., larger operational range, as the wider bandwidth difference among signals eases the differentiation between them, thus presenting a lower error rate. On the opposite, slope values are steeper for worse approaches, i.e., those with shorter operational range, as the smaller bandwidth difference between signals conduct to high error rate in the differentiation process. This could be improved with the use of more selective filters, but that increases the complexity of the circuitry.

A key component in the WuR system proposed and evaluated in this paper consists in the ability of the transmitter to change the signal bandwidth on a per-packet basis, which is a common feature in modern Wi-Fi radios. In this way, we have verified that already existing devices, such as TI CC3200 [29], allow this functionality. Specifically, the TI CC3200 device, which supports IEEE 802.11n specification, is able of switching between signal bandwidths of 20 MHz and 40 MHz for consecutive transmissions. However, in this case, we observed that an additional delay is introduced due to this switching function, which increases the inter-frame time above the SIFS value. In our experiments, this inter-frame time has been observed to be between 100 and 200 µs. Thus, further research needs to be conducted in the real implementation of the proposed WuR system, which is out of the scope of the present work and is considered as part of our future work.

We have to note that the operational ranges provided by the proposed IEEE 802.11-based WuR solution are smaller than the maximum achievable distances employing the IEEE 802.11 primary interfaces for data exchange. Ideally, having the same maximum operational ranges for both primary and WuR interfaces is desirable. Having a shorter reach in the secondary radio limits the applicability of the solution to shorter distances, but the problem of having different ranges in both radios becomes more significant when the secondary radio exceeds the maximum distance achieved by the primary (the system is able to wake up the primary radio, but then the primary radio cannot operate effectively). However, it is worth mentioning that using the fastest MCSs available for the different IEEE 802.11 amendments, the distances corresponding to the communication through IEEE 802.11 primary interfaces will be reduced to ranges around, or less than, the values provided by the WuR solution exposed. Under those conditions, the range of both WuR and primary interface data communications will be aligned. Certainly, the maximization of the WuR operational range is a relevant issue in this topic, and further work is planned to be undertaken in this direction.

## 6. Conclusions and Future Work

In this paper, we have proposed a WuR system based on the transmission of legacy frames by an IEEE 802.11 standard-compliant transmitter during a TXOP period. We have performed system validation through the Matlab software tool and shown the feasibility of the solution proposed, considering legacy IEEE 802.11 transmitters and different propagation models from TGn/ac. The proposed solution includes the design of a simple receiver. It provides operational ranges comprised between 18 m and 50 m using one bit per symbol (17.26 kbps, 16.14 kbps, and 13.58 kbps for IEEE 802.11n, 11ac, and 11ax, respectively), and between 12 m and 30 m for two bits per symbol (32.81 kbps and 27.17 kbps for IEEE 802.11ac and 11ax, respectively), depending on the propagation conditions. The bit rate achieved is higher than for other WuR solutions based on the transmission of legacy IEEE 802.11 frames. Although similar to other solutions, the operational range is short if we assume the requirement of having a range comparable to the primary radio. Both ranges converge when the fastest MCSs are being used in communications through the primary radio. However, further research is necessary to be conducted. In this sense, the next step in our future work is to study more efficient WuRx designs. Additionally, the WuR system presented includes intrinsic protection in front of other transmissions (other WuCs and IEEE 802.11 legacy transmissions) due to the usage of the standard-compliant TXOP mechanism. Besides, we have verified the feasibility of this solution using off-the-shelf Wi-Fi devices by looking at their capability to change the transmission bandwidth on a per-packet basis. However, experiments showed an additional delay in the inter-frame time, thus slightly reducing the effective bit rate of the generated WuC. As part of our future work, we plan to carry out further research in a practical implementation of the proposed and validated WuR solution presented in this paper.

## Figures and Tables

**Table 1:** Codification scheme.

Signal Bandwidth	Codification Scheme
20 MHz	00
40 MHz	01
80 MHz	11
160 MHz	10

## Data Availability

Not applicable.
